# 改良的QuEChERS-超高效液相色谱-三重四极杆质谱法检测银耳和木耳中米酵菌酸

**DOI:** 10.3724/SP.J.1123.2021.06013

**Published:** 2021-12-08

**Authors:** Pan ZOU, Shengxing DUAN, Xizhou HU, Dan ZHENG, Zhenzhen XIA, Hong XIA, Xitian PENG

**Affiliations:** 湖北省农业科学院农业质量标准与检测技术研究所, 农产品营养品质与安全湖北省重点实验室, 湖北 武汉 430064; Institute of Agricultural Quality Standards and Testing Technology Research, Hubei Academy of Agricultural Science, Hubei Key Laboratory of Nutritional Quality and Safety of Agro products, Wuhan 430064, China; 湖北省农业科学院农业质量标准与检测技术研究所, 农产品营养品质与安全湖北省重点实验室, 湖北 武汉 430064; Institute of Agricultural Quality Standards and Testing Technology Research, Hubei Academy of Agricultural Science, Hubei Key Laboratory of Nutritional Quality and Safety of Agro products, Wuhan 430064, China; 湖北省农业科学院农业质量标准与检测技术研究所, 农产品营养品质与安全湖北省重点实验室, 湖北 武汉 430064; Institute of Agricultural Quality Standards and Testing Technology Research, Hubei Academy of Agricultural Science, Hubei Key Laboratory of Nutritional Quality and Safety of Agro products, Wuhan 430064, China; 湖北省农业科学院农业质量标准与检测技术研究所, 农产品营养品质与安全湖北省重点实验室, 湖北 武汉 430064; Institute of Agricultural Quality Standards and Testing Technology Research, Hubei Academy of Agricultural Science, Hubei Key Laboratory of Nutritional Quality and Safety of Agro products, Wuhan 430064, China; 湖北省农业科学院农业质量标准与检测技术研究所, 农产品营养品质与安全湖北省重点实验室, 湖北 武汉 430064; Institute of Agricultural Quality Standards and Testing Technology Research, Hubei Academy of Agricultural Science, Hubei Key Laboratory of Nutritional Quality and Safety of Agro products, Wuhan 430064, China; 湖北省农业科学院农业质量标准与检测技术研究所, 农产品营养品质与安全湖北省重点实验室, 湖北 武汉 430064; Institute of Agricultural Quality Standards and Testing Technology Research, Hubei Academy of Agricultural Science, Hubei Key Laboratory of Nutritional Quality and Safety of Agro products, Wuhan 430064, China; 湖北省农业科学院农业质量标准与检测技术研究所, 农产品营养品质与安全湖北省重点实验室, 湖北 武汉 430064; Institute of Agricultural Quality Standards and Testing Technology Research, Hubei Academy of Agricultural Science, Hubei Key Laboratory of Nutritional Quality and Safety of Agro products, Wuhan 430064, China

**Keywords:** QuEChERS, 超高效液相色谱-串联质谱, 米酵菌酸, 银耳, 木耳, QuEChERS, ultra-high performance liquid chromatography-tandem mass spectrometry (UHPLC-MS/MS), bongkrekic acid, tremella, auricularia auricular

## Abstract

采用改良的QuEChERS方法,结合超高效液相色谱-串联质谱(UHPLC-MS/MS)建立了银耳和木耳中米酵菌酸含量测定的分析方法。对QuEChERS方法的提取、净化条件进行了优化,发现提取液中乙酸含量对米酵菌酸的提取效率影响很大,最终采用5%(v/v)乙酸乙腈为提取溶剂,盐析分层,再用200 mg C18分散固相萃取净化。对UHPLC-MS/MS分析条件也进行了优化,以含0.01%(v/v)甲酸、0.05%(v/v)氨水的水溶液和甲醇为流动相,在Waters HSS T3柱(100 mm×2.1 mm, 1.8 μm)上分离,以电喷雾电离、多反应监测负离子模式进行检测。在优化好的条件下,银耳和木耳中米酵菌酸检测的基质效应分别为-6.3%和-11.5%,表明该方法具有很好的净化效果,样品基质不会对米酵菌酸的检测产生影响。进一步对方法学进行了考察,在1~200 μg/L范围内,线性方程回归系数的平方(*R*^2^)大于0.999,以信噪比的3倍和10倍计算的方法检出限和定量限分别为0.15 μg/kg和0.5 μg/kg。银耳中3种添加水平0.5、10、50 μg/kg下的加标回收率为92.4%~102.6%,日内和日间相对标准偏差(RSD)分别为4.3%~4.9%和3.2%~3.5%;木耳的加标回收率为89.6%~102.3%,日内和日间RSD分别为2.4%~9.5%和3.6%~4.1%,表明该方法具有很好的准确度和精密度。最后,将该方法应用于实际样品中米酵菌酸的分析,取得了很好的效果。该工作为银耳和木耳中米酵菌酸的风险防控提供了一种有效的检测技术。

## Abstract (en)

An improved QuEChERS (quick, easy, cheap, effective, rugged, safe) method, combined with ultra-high performance liquid chromatography-tandem mass spectrometry (UHPLC-MS/MS), was used to determine bongkrekic acid (BA) in tremella and auricularia auricular. BA is a fat-soluble, fatal bacterial toxin produced by the aerobic gram-negative bacteria Burkholderia gladioli pathovar cocovenenans. Tremella and auricularia auricular, which have rich nutritional values, are traditional edible fungi in China that are very popular among Chinese consumers. However, tremella and auricularia auricular are easily contaminated by BA during storage and foaming, and this poses a great threat to food safety and human health. Therefore, establishing a rapid and efficient analysis method for detecting BA in tremella and auricularia auricular is of considerable significance. However, the BA concentration in the actual sample is very low, and the matrices of tremella fuciformis and auricularia auricular are very complex. Thus, it is necessary to employ appropriate sample pretreatment technology to extract and purify BA from tremella and auricularia auricular samples prior to instrumental analysis. In this study, the QuEChERS method, combined with UHPLC-MS/MS, was used to detect BA in tremella and auricularia auricular. The key parameters, such as extraction solvent, extraction method, and adsorbent used for cleanup, were optimized to obtain high extraction efficiency. The content of acetic acid in the extraction solution strongly influenced the extraction efficiency of BA, and acetonitrile with 5%(v/v) acetic acid was determined to be the optimum extraction solvent. After salting out, the acetonitrile extract was purified by dispersive solid phase extraction using 200 mg C18 as a cleanup adsorbent. The sample was then separated on a Waters HSS T3 column (100 mm×2.1 mm, 1.8 μm), using a water solution containing 0.01% (v/v) formic acid and 0.05% (v/v) ammonia and methanol as mobile phases. MS analysis was performed using an electrospray ionization source in the negative and multiple reaction monitoring (MRM) modes. Under the optimized conditions, the matrix effects of UHPLC-MS/MS in tremella and auricularia auricular were -6.3% and -11.5%, respectively; this indicated that the method had a significant purification effect, and the sample matrix did not affect the MS detection of BA. Further study showed that in the concentration range of 1-200 μg/L, the square of the regression coefficient of the linear equation (*R*^2^) was greater than 0.999. The limit of detection (LOD) and limit of quantitation (LOQ) were 0.15 μg/kg and 0.5 μg/kg, respectively. The average recoveries in samples spiked with 0.5, 10, and 50 μg/kg BA in tremella ranged from 92.4% to 102.6%, and the intra-day and inter-day relative standard deviations (RSDs) were 4.3%-4.9% and 3.2%-3.5%, respectively. For auricularia auricular, the average recoveries ranged from 89.6% to 102.3%, and the intra-day and inter-day RSDs were 2.4%-9.5% and 3.6%-4.1%, respectively. These results indicate that the proposed method has satisfactory sensitivity, accuracy, and precision. Finally, the method showed good performance when applied to the analysis of real samples. Compared with other reported methods, the LOD and LOQ of our proposed method were lower, with satisfactory recovery and precision. Taken together, this study provides an effective detection technology for the monitoring and risk control of BA in tremella and auricularia auricular.

米酵菌酸(bongkrekic acid, BA)是一种脂溶性细菌毒素,是由食品中常见的致病菌椰毒假单胞菌产生的,谷物、发酵的米面制品、银耳和木耳等食品最易受到该病菌的污染^[1]^。研究表明,米酵菌酸对线粒体腺嘌呤核苷酸转移酶(adenine nucleotide translocase, ANT)的活性产生抑制,从而破坏线粒体中三磷酸腺苷(adenosine triphosphate, ATP)的合成,导致细胞的凋亡^[2,3]^。因此,人体摄入经米酵菌酸污染的食品后,可能会损伤肝、肾、心、脑等重要器官的细胞,很容易导致食物中毒^[4]^。2020年黑龙江鸡西市的“酸汤子”、2015年莫桑比克的椰子制品、2014年云南文山汤圆等食物中毒事件都导致了严重的死亡事件,经查这些食物中毒都是由米酵菌酸引起的^[1]^。银耳和木耳是我国传统的食用菌,其营养价值丰富,深受我国消费者的喜爱。然而,银耳和木耳在储藏和泡发过程中易受米酵菌酸的污染,给食品安全造成了极大的威胁^[5,6]^。因此,建立银耳和木耳中米酵菌酸快速、高效的分析方法具有重要的意义。

米酵菌酸是一种共轭的不饱和脂肪酸(见图1),常见的检测方法有高效液相色谱-紫外检测法^[7,8]^和高效液相色谱-质谱法^[1,4,6,9,10]^。与传统的紫外检测器相比,超高效液相色谱-串联质谱法(UHPLC-MS/MS)检测灵敏度高和准确度更好,其在多反应监测模式下可以通过目标物的保留时间、母离子质荷比以及碎裂反应对目标分析物进行定性,分析方法具有更好的特异性,可以大大减少基质的干扰,降低假阳性分析结果的出现^[11]^。此外,米酵菌酸在样品中的浓度很低,银耳和木耳基质复杂,采取适当的样品前处理技术对其进行提取净化非常有必要。目前,应用最广泛的米酵菌酸前处理方法是固相萃取(SPE),其主要采用混合模式阴离子交换固相萃取柱,通过混合模式的反相和阴离子交换作用实现米酵菌酸的萃取富集^[1,4,7,8]^。SPE方法的结果稳定,回收率较高,然而SPE的上样、清洗、解吸等步骤较为繁琐,耗时较长,SPE柱的成本也较高。QuEChERS方法由美国农业部2003年首次提出,其具有快速、简单、便宜、有效和安全的特点,在食品农药残留分析中展现了极大的应用前景^[12,13,14]^。近年来,QuEChERS方法在食品中真菌毒素如黄曲霉毒素、赭曲霉毒素、玉米赤霉烯酮、伏马毒素等的分析中同样取得了很好的效果^[15,16,17]^。到目前为止,尚未检索到QuEChERS方法用于银耳和木耳中米酵菌酸的检测。

**图1:**
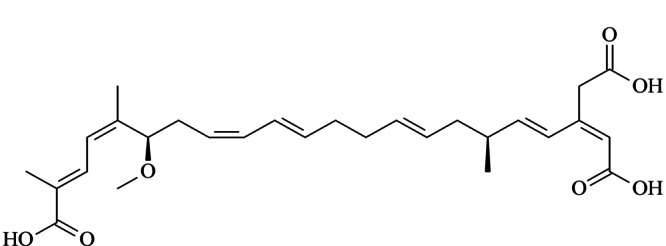
米酵菌酸的化学结构

因此,本文采用改良的QuEChERS前处理方法,对提取溶剂、提取方式、净化材料等进行了详细的优化,结合UHPLC-MS/MS的分离检测,建立了银耳和木耳中米酵菌酸的快速分析方法,旨在为食品中米酵菌酸的监测和风险防控提供一种有效的检测技术。

## 1 实验部分

### 1.1 仪器、试剂及材料

LC-30AD液相色谱仪(日本Shimadzu公司),Qtrap 4500三重四极杆质谱仪(AB SCIEX公司);数据采集由Analyst^@^工作站完成,数据处理由MultiQuant分析软件完成。TDL-6C台式大容量离心机(上海安亭科学仪器厂);MS 3型涡旋仪(德国IKA公司);T25型匀浆搅拌器(德国IKA公司);KQ-500B型超声波清洗器(昆山超声仪器有限公司);氮吹仪(美国Organomation公司);Milli-Q超纯水处理仪(美国Millipore公司);ME203E型电子天平(上海梅特勒-托利多仪器有限公司)。

米酵菌酸标准品(纯度大于99%,上海宏叶生物科技有限公司);色谱纯甲醇、乙腈(德国默克公司),色谱纯甲酸(Fisher Scientific公司),色谱纯乙酸(美国JT. Baker公司)。石墨化炭黑GCB(120~400目)、C18(50 μm)和PSA(40~60 μm)购自天津博纳艾杰尔科技有限公司。尼龙针头过滤器(0.22 μm)购自天津市津腾实验设备有限公司。所有的银耳和木耳样品购自湖北省菜市场和超市。

### 1.2 标准溶液的制备

标准储备液:将0.1 mg的米酵菌酸标准品溶于10 mL甲醇中得到10 mg/L的标准储备液,置于-20 ℃冰箱保存。再吸取10 mg/L米酵菌酸标准溶液1 mL置于10 mL容量瓶中,用甲醇定容至10 mL,得到1 mg/L的标准储备液,置于4 ℃冰箱保存备用。

标准溶液:使用银耳和木耳样品提取净化后的溶液和50%(v/v)甲醇水溶液分别配制1.0、2.0、5.0、10.0、50.0、100.0和200.0 μg/L的基质标准溶液和溶剂标准溶液。

### 1.3 样品前处理

称取2.0 g干燥粉碎的银耳样品于50 mL离心管中,加入10 mL水和10 mL 5%(v/v)乙酸乙腈,涡旋提取1 min,加入6.0 g无水硫酸镁和1.5 g醋酸钠,涡旋1 min, 4 000 r/min离心5 min,取8 mL上清液加入到装有200 mg C18和900 mg无水硫酸镁的10 mL离心管中,涡旋净化1 min后离心,收集5 mL上清液,氮气吹干,用1 mL 50%(v/v)甲醇水溶液复溶,经0.22 μm的尼龙滤膜过滤后,UHPLC-MS/MS分离检测。

### 1.4 分析条件

色谱条件 色谱柱:Waters ACQUITY UPLC@HSS T3柱(100 mm×2.1 mm, 1.8 μm)(Waters,爱尔兰);流动相:A为含0.01%(v/v)甲酸和0.05%(v/v)氨水的水溶液,B为甲醇。色谱线性梯度程序:0~3 min, 40%B~90%B; 3~3.5 min, 90%B; 3.51~5.50 min, 40%B。流速:0.3 mL/min;柱温:40 ℃;样品室温度:15 ℃;进样体积:5 μL。

质谱条件 离子源:电喷雾(ESI);扫描方式:多反应监测(MRM)负离子扫描模式;离子化电压(IS): -4500 V;离子源温度:550 ℃;气帘气(CUR): 172.4 kPa(25 psi);喷雾气(GS1): 379.2 kPa(55 psi);辅助加热气(GS2): 379.2 kPa(55 psi);碰撞气:Medium。定量离子对*m/z* 485.3>441.0,碰撞电压为-14.6 V;定性离子对*m/z* 485.3>397.1,碰撞电压为-23.6 V。

## 2 结果与讨论

### 2.1 色谱-质谱条件的优化

米酵菌酸是长链的不饱和三元羧酸,在电喷雾质谱的负离子模式下具有较高的灵敏度。首先对质谱条件进行了优化,在Q1全扫模式下对母离子进行了扫描,发现[M-H]^-^(*m/z*, 485.3)的响应值最高。随后,在子离子扫描模式下对定性和定量离子进行了扫描,发现母离子会连续产生两个[CO_2_]的中性丢失,产生*m/z*为441.0和397.1的子离子,与文献^[9]^的报道一致。进一步对定性和定量离子对的碰撞电压进行了优化,选择灵敏度更高的*m/z* 485.3>441.0为定量离子对,碰撞电压为-14.6 V; *m/z* 485.3>397.1为定性离子对,碰撞电压为-23.6 V。

对液相色谱条件进行了优化,当水相采用纯水时,米酵菌酸的峰形很差,流动相中加入0.01%甲酸和0.05%氨水可以有效地改善米酵菌酸的峰形。同时,对有机相的起始比例从10%到50%进行了考察,发现40%的甲醇作为起始比例,3 min内升到90%,米酵菌酸的保留时间为2.5 min,保留时间较短,同时样品基质对分离没有干扰。最后,对进样体积和柱温等条件进行了优化,优化好的色谱条件如1.4节所述,米酵菌酸的多反应监测色谱图见图2。

**图2:**
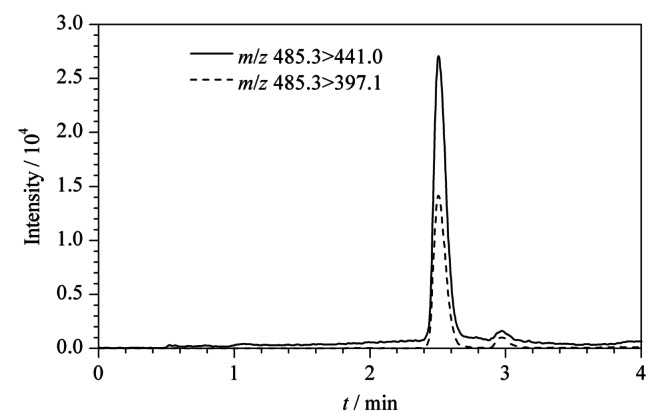
20 μg/L米酵菌酸的多反应监测色谱图

### 2.2 样品前处理条件的优化

为了获得较高的回收率,我们优化了一系列可能影响萃取效率的条件:净化材料的用量、提取方式、提取溶剂乙酸含量等。我们以空白加标的银耳样品(20 μg/L)对改进的QuECHERs方法进行了优化,每个条件平行处理3次。

2.2.1 样品与水比例的优化

对于水含量很低的样品,为了提高目标物的提取效率,通常会往样品中加入一定量的水^[18]^。银耳和木耳晒干后含水量很低,泡发不充分会影响样品的提取效果,本实验考察了水加入量对米酵菌酸提取的影响,在2.0 g银耳中分别加入了4、5、6、8和10 mL水,结果显示,当水加至10 mL时能够使银耳充分泡发。因此我们选择加入10 mL的水。

2.2.2 提取方式的优化

由于银耳基质复杂,且加水泡发后体积膨大,需要一个合适的提取方式来提取目标物。我们分别采用不同的提取方式:超声、涡旋和匀浆提取2 min,考察了3种方法对样品中米酵菌酸提取效率的影响。结果表明,涡旋提取的回收率为89.5%,匀浆提取的为74.1%,但超声提取的回收率较前两者低,为46.5%。考虑到匀浆提取搅拌头每次匀浆后需清洗,加大了工作量和时长,因此我们选择涡旋提取。

2.2.3 提取溶剂的优化

乙腈有很好的细胞穿透性,是最常用的QuEChERS提取溶剂,而本实验的目标物米酵菌酸有3个羧酸,加入适量的乙酸能破坏银耳基质中金属离子与米酵菌酸之间的络合作用,同时会使得米酵菌酸的羧基质子化,极性变弱,增大了米酵菌酸在乙腈中的溶解度。我们考察了不同乙酸含量的乙腈,如图3所示,当提取液为纯乙腈时,米酵菌酸回收率不到10%,随着乙酸的体积分数从0%增加到5%,回收率快速增加,5%的乙酸提取时回收率可以达到90%以上;当乙酸的体积分数继续增加至10%,回收率降低,过高的酸性可能会影响米酵菌酸的稳定性。因此我们选择的提取溶剂是含5%乙酸的乙腈。

**图3:**
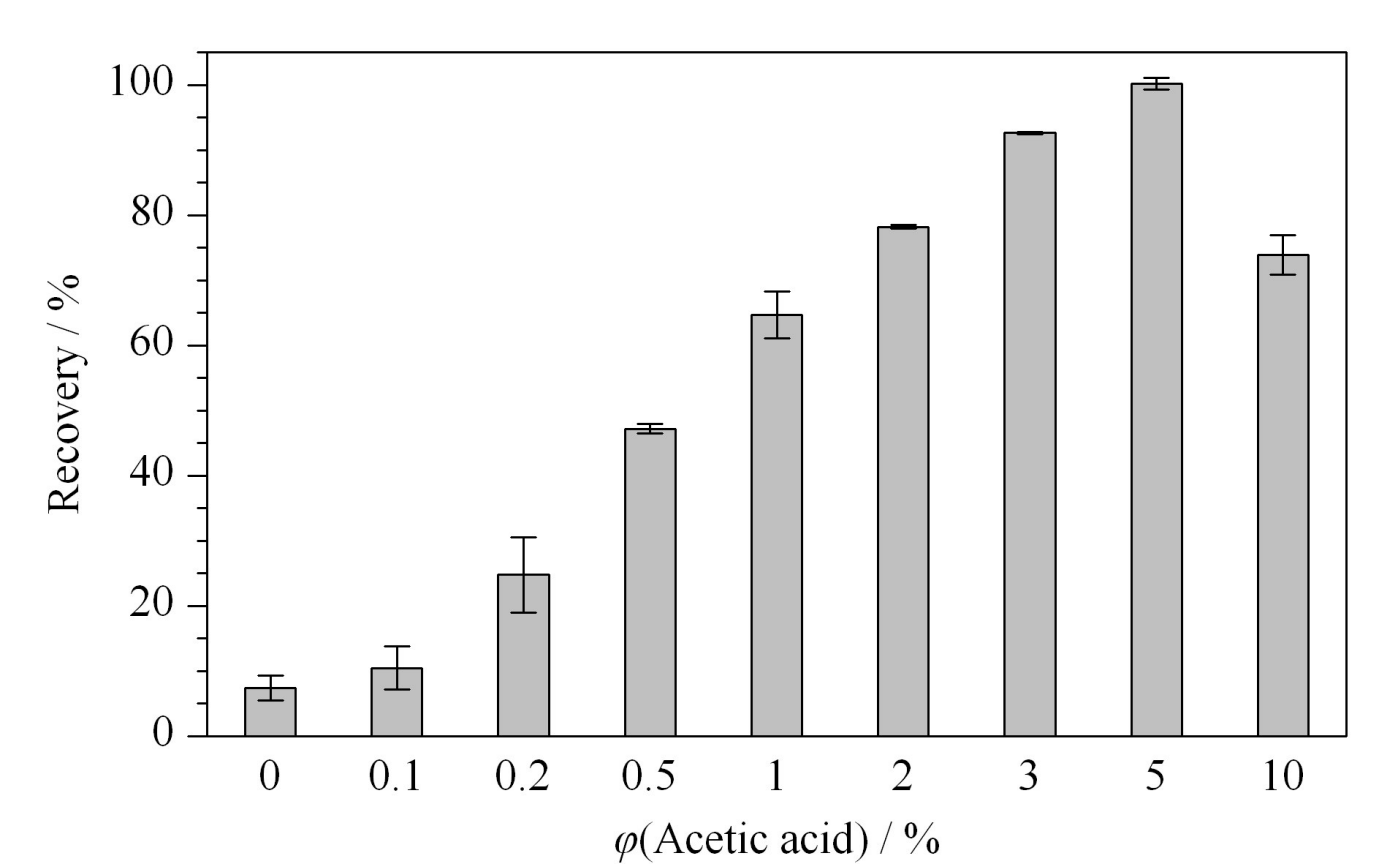
乙酸的体积分数对银耳中米酵菌酸回收率的影响(*n*=3)

2.2.4 吸附剂的优化

本实验选取了3种常用的QuEChERS吸附剂:PSA、C18和GCB,考察了吸附剂种类和用量对米酵菌酸萃取效率的影响。在实验过程中,我们发现虽然GCB有去除银耳提取液中色素等干扰物的能力,但同时它会吸附疏水的米酵菌酸,从而减少回收率;而PSA是碱性的,会与酸性的米酵菌酸相互作用而降低回收率。C18极性较弱,可以除去一部分的脂类干扰物,因此本实验中我们比较了不同用量的C18吸附剂,当吸附剂含量由100 mg增至200 mg时,回收率逐渐增加,由61.1%升至70.1%;当吸附剂继续增至250 mg,回收率缓慢降为66.3%。因此我们选择200 mg的C18净化银耳提取液。

### 2.3 方法学考察

2.3.1 基质效应

在HPLC-MS分析中,样品中的基质可能会增强或抑制分析物的离子化效率,进而影响其在仪器上的响应信号,引起实验结果的偏差,因此需要对本实验进行基质效应评估。本研究采用以下公式^[19]^评价方法的基质效应:

$\mathrm{基质效应}=\left(\frac{\mathrm{基质标准曲线的斜率}}{\mathrm{溶剂标准曲线的斜率}}-1\right)\times 100\%$

一般来说,基质效应在-20%~20%为弱基质效应;在-50%~-20%和20%~50%为中等基质效应;小于-50%或大于50%则为强基质效应。结果表明,银耳和木耳中米酵菌酸的基质效应分别为-6.3%和-11.5%,由此可见,经过QuEChERS提取净化后,样品的基质效应较弱,样品基质对分析物的影响较小。

2.3.2 线性关系、检出限和定量限

由于本实验中银耳和木耳样品的基质效应很弱,因此,我们采用溶剂标准曲线对该实验的线性关系、检出限、定量限以及精密度等进行了考察,验证该方法在实际应用中的可行性。

溶剂标准曲线是50%(v/v)甲醇水溶液配制成1~200 μg/L的一系列浓度的标准溶液,按1.4节方法测定,米酵菌酸在1~200 μg/L的范围内的线性方程为*y*=2937.6*x*+8204.2(*y*为峰面积,*x*为米酵菌酸的质量浓度(μg/L)),线性关系良好,回归系数的平方(*R*^2^)大于0.999。以信噪比的3倍和10倍计算方法的检出限和定量限分别为0.15 μg/kg和0.5 μg/kg。

2.3.3 回收率和精密度

为了评估该方法的准确性,我们分别在银耳和木耳样品基质中添加了LOQ(0.5 μg/kg)、中(10 μg/kg)、高(50 μg/kg)3个浓度的目标分析物,同时配制6组平行样品进行试验,得到的检测结果用以计算日内相对标准偏差(RSD);以连续6天单独配制的样品进行实验,得到的检测结果用以计算日间RSD。结果如表1和表2所示,银耳方法的加标回收率在92.4%~102.6%之间。日内RSD在4.3%~4.9%,日间RSD的范围在3.2%~3.5%;木耳方法的加标回收率在89.6%~102.3%之间。日内RSD在2.4%~9.5%,日间RSD的范围在3.6%~4.1%。以上结果表明该方法的重现性较好。

**表1:** 米酵菌酸在银耳和木耳样品中低、中、高水平下的加标回收率和RSD (*n*=6)

Sample	0.5 μg/kg			10 μg/kg			50 μg/kg	
	Recovery/%	RSD/%		Recovery/%	RSD/%		Recovery/%	RSD/%
Tremella	102.6	4.7		98.6	4.9		92.4	4.3
Auricularia auricular	89.6	7.7		95.6	2.4		102.3	9.5

**表2:** 米酵菌酸测定的日内、日间精密度(n=6)

Sample	Intra-day precision/%				Inter-day precision/%		
	0.5 μg/kg	10 μg/kg	50 μg/kg		0.5 μg/kg	10 μg/kg	50 μg/kg
Tremella	4.7	4.9	4.3		3.5	3.3	3.2
Auricularia auricular	7.7	2.4	9.5		4.1	3.7	3.6

2.3.4 在实际样品分析中的应用

为了考察方法的普适性,我们将该方法应用于武汉市场上10种银耳和10种木耳中米酵菌酸的检测。在这10种银耳中未检测到阳性样品,而木耳中检出一个阳性样品,含量为21.48 μg/kg。说明方法具有普适性,可应用于银耳和木耳中米酵菌酸的检测。

2.3.5 方法比较

对本文建立的方法与文献报道的方法进行了比较。米酵菌酸分子含有3个羧酸根,文献通常采用有机溶剂提取后混合型阴离子交换柱富集净化^[6,7,20]^。从样品前处理过程来看,本文的QuEChERS方法较为简单,避免了SPE方法繁琐的提取、上样、清洗和解吸过程,样品提取净化成本低,基质效应弱,结合UHPLC-MS/MS进行检测,进一步提高了检测结果的准确度和灵敏度。同时,与其他检测方法^[6,7,20]^相比,本方法的检出限和定量限更低(0.15 μg/kg和0.5 μg/kg),其他方法的检出限和定量限最低分别为0.5 μg/kg和2 μg/kg。同时,本方法的回收率和精密度与其他方法相当,在实际样品大批量分析中具有很好的应用潜能。

## 3 结论

本文将QuEChERS方法结合UHPLC-MS/MS用于银耳和木耳中米酵菌酸的检测,样品前处理过程快速、简单、有效,同时具有灵敏度高、准确度好的优点,为食品中米酵菌酸的风险防控提供了一种有效的检测技术。
